# Outcomes of 1.3 million patients undergoing percutaneous coronary intervention according to the presence of cancer and atrial fibrillation: a retrospective study

**DOI:** 10.3325/cmj.2024.65.405

**Published:** 2024-10

**Authors:** Sedralmontaha Istanbuly, Andrija Matetić, Vijay Bang, Kamal Sharma, Harsh Golwala, Babikir Kheiri, Mohammed Osman, Pooja Swamy, Aditya Bharadwaj, Mamas Mamas

**Affiliations:** 1 Faculty of Medicine, University of Aleppo, Aleppo, Syria; 2 Department of Cardiology, University Hospital of Split, Split, Croatia; 3 Lilavati Hospital and Research Center, Mumbai, India; 4 U.N. Mehta ICRC, B. J. Medical College, Ahmedebad, India; 5 Knight Cardiovascular Institute, Oregon Health & Science University, Portland, OR; 6 Section of Cardiac Electrophysiology, University of California San Francisco, San Francisco, CA; 7 Section of Cardiovascular Medicine, Yale-New Haven Hospital, Yale School of Medicine, New Haven, CT; 8 Division of Cardiology, Loma Linda University, Loma Linda, CA; 9 Keele Cardiovascular Research Group, Keele University, Newcastle, United Kingdom

## Abstract

**Aim:**

To evaluate outcomes after percutaneous coronary intervention (PCI) in patients with cancer and atrial fibrillation (AF).

**Methods:**

Data of all adult discharges undergoing PCI between October 2015 and December 2018 were obtained from the National Inpatient Sample (NIS) database. Adjusted odds ratios (aOR) of adverse complications were calculated using binominal logistic regression.

**Results:**

1 387 320 patients were detected, out of which 15.4% had AF but no cancer, 1.9% had cancer but no AF, and 0.6% had both cancer and AF. Compared with cancer patients without AF, those with AF had a greater aOR of mortality (aOR 1.20, 95%CI 1.08-1.33), major adverse cardiac and cerebrovascular events (MACCE) (aOR 1.18, 95%CI 1.07-1.29), and bleeding (aOR 1.23, 95%CI 1.08-1.39). However, the risk of ischemic stroke was similar between the two groups. Patients with solid cancer and AF had a higher aOR for all outcomes, including mortality (aOR 1.28, 95%CI 1.09-1.50), MACCE (aOR 1.37, 95%CI 1.19-1.57), ischemic stroke (aOR 1.48, 95%CI 1.10-1.99), and bleeding (aOR 1.66, 95%CI 1.39-1.98) compared with the solid cancer group without AF. In patients with hematological cancer, AF was associated only with significantly increased risk of mortality (aOR 1.40, 95%CI 1.16-1.70) and MACCE (aOR 1.26, 95%CI 1.06-1.49).

**Conclusions:**

The presence of AF in solid cancer patients increases the risk of mortality, MACCE, stroke, and major bleeding, while in the setting of hematological cancer it is only associated with a higher risk of mortality and MACCE.

Cardiovascular disease (CVD) and cancer represent the two leading causes of death worldwide (1,2). Among the 19 million patients who died due to CVD in the US between 1999 and 2019, 13.6% were diagnosed with concomitant cancer (3). Many patients with concomitant coronary artery disease (CAD) and cancer undergo percutaneous coronary intervention (PCI), representing a high-risk population. Among ~ 6.5 million patients undergoing PCI, 5.8% had previous cancer diagnoses, and 1.8% had current cancer diagnoses, with prostate, breast, colon, and lung cancer being the most prevalent (4). In addition, a meta-analysis reported that patients with concomitant cancer undergoing PCI had higher one-year mortality than non-cancer patients (5).

Atrial fibrillation (AF) is prevalent in cancer patients, who have around 50% increased risk of AF compared with non-cancer patients (6). Patients with AF have a higher mortality risk when undergoing PCI than those without AF (7). In addition, cancer patients with AF could have an increased risk of thrombotic events and major bleeding compared with their non-cancer counterparts (8,9). However, there is limited data regarding PCI outcomes among patients with concomitant cancer and AF. Previous studies explored the clinical outcomes of PCI among patients with cancer only, with AF only, or outcomes of AF and cancer only. Therefore, this study aimed to determine the outcomes of PCI in patients with AF in non-cancer and cancer groups. Given that patients with cancer represent a heterogeneous cohort with varying outcomes of PCI depending on the type of cancer (4), we sought to further analyze outcomes based on cancer type.

## Patients and methods

### Data source

The National Inpatient Sample (NIS) is the largest publicly available all-payer inpatient care database, which collects anonymized data on more than seven million hospital discharges in the United States (US) each year. It is developed by the Healthcare Cost and Utilization Project (HCUP) and sponsored by the Agency for Healthcare Research and Quality. The NIS covers a 20-percent stratified sample of all US community hospitals, which approximates about 97% of the US population (10). The analyses in this study are exempt from institutional or ethics committee review as all data are publicly available, de-identified, and anonymized.

### Study design and population

Data of all adult (≥18 years) hospitalized patients who underwent at least one PCI between October 2015 and December 2018 were obtained from the NIS database and analyzed. The study sample was stratified by the cancer status and AF presence into four groups: non-cancer patients without AF, non-cancer patients with AF, cancer patients without AF, and cancer patients with AF. Cancer patients were further stratified by the cancer type into solid cancer (colorectal, lung, breast, and prostate cancer), hematological cancer, and other cancer groups. We identified patients’ characteristics, study groups, and clinical outcomes using the International Classification of Diseases, 10th revision and Clinical Classification Software codes (Supplemental Table 1(Supplementary Table 1)). We excluded patients with missing data for the following variables: age, sex, length of stay, primary expected payer, mortality status, elective admission, total charges, and weekend admission (n = 19 745 [1.4%]). Supplemental Figure 1(Supplementary figure 1) shows the study protocol including the steps of selecting the study sample.

### Study outcomes

The primary outcome was in-hospital all-cause mortality. Secondary outcomes included in-hospital major adverse cardiovascular and cerebrovascular events (MACCE), acute ischemic stroke, and major bleeding. MACCE was defined as a composite of all-cause mortality, acute ischemic stroke, or transient ischemic attack, and re-infarction. Major bleeding was defined as intracranial or gastrointestinal hemorrhage. CHA_2_DS_2_VASc risk score was composed of the following components: congestive heart failure, arterial hypertension, age cut-offs (65-75 and ≥75 years), diabetes mellitus, previous stroke, vascular disease, and sex category (11).

### Statistical analysis

Categorical data are presented as percentages (numbers), and continuous data are presented as medians (interquartile ranges). The χ^2^ test was used to compare categorical variables, whereas the Kruskal-Wallis and Mann-Whitney tests were used to compare continuous variables where appropriate. Adjusted odds ratios (aOR) and 95% confidence intervals (CI) of clinical outcomes were obtained using binominal multivariable logistic regression analysis. The analysis was adjusted for the following variables: age, sex, hospital bed size, hospital location/teaching status, hospital region, weekend admission, primary expected payer, dyslipidemia, smoking, dementia, thrombocytopenia, hypertension, diabetes, anemia, chronic renal failure, chronic lung disease, coagulopathy, liver disease, previous cerebrovascular accident, previous myocardial infarction, previous PCI, and previous coronary artery bypass graft (CABG). The following variables were removed from the multivariable model in the CHA_2_DS_2_VASc sensitivity analysis due to being part of the CHA_2_DS_2_VASc risk score: hypertension, diabetes, previous cerebrovascular accident, previous myocardial infarction, previous PCI, and previous CABG. We considered a *P* value less than 0.05 statistically significant. All analyses were weighted and performed with SPSS 25 software (IBM Corp., Armonk, NY, USA).

## Results

Out of 1 387 320 included patients, 2.5% had a cancer diagnosis and 16.0% had an AF diagnosis. Furthermore, 82.1% had neither cancer nor AF; 15.4% had AF but no cancer; 1.9% had cancer but no AF; and 0.6% had both cancer and AF (Table 1). Solid cancer was the most common type of cancer, amounting to 40% of total cancer patients (prostate: 14.4%; lung: 13.5%; colorectal: 6.7%; breast: 5.4%), followed by hematological cancer (30.9%) and other cancer (29.1%) (Supplemental Figure 2(Supplementary figure 2)).

**Table 1:** Patient characteristics according to cancer and atrial fibrillation diagnosis*

Characteristics	Non-cancer group		*P*-value	Cancer group		*P*-value
	without AF (82.1%)	with AF (15.4%)		without AF (1.9%)	with AF (0.6%)	
Number of hospitalizations	1 139 230	213 595		26 245	8250	
Age (years), median (IQR)	63 (55, 72)	73 (65, 81)	<0.001	70 (63, 77)	76 (69, 82)	<0.001
Female sex, %	33.1	33.5	<0.001	31.4	26.2	<0.001
Race, %			<0.001			<0.001
White	74.9	83.5		80.7	85.3	
Black	10.6	6.4		9.9	5.2	
Hispanic	7.8	5.4		5.1	3.8	
Other	6.6	4.8		4.2	5.7	
Elective admission, %	9.5	11.2	<0.001	11.6	12.8	0.002
Weekend admission, %	23.9	22.7	<0.001	22.6	21.7	0.077
Primary expected payer, %			<0.001			<0.001
Medicare	49.0	75.0		70.6	82.2	
Medicaid	10.3	4.8		6.3	2.4	
private insurance	31.9	15.8		19.4	13.0	
self-pay	5.3	2.0		1.4	0.7	
no charge	0.5	0.2		0.2	0.1	
other	3.1	2.2		2.2	1.7	
Median household income (percentile), %			<0.001			<0.001
0-25th	30.2	28.4		27.8	24.6	
26th-50th	27.7	27.9		26.4	27.0	
51st-75th	23.8	24.2		24.4	25.7	
76th-100th	18.3	19.5		21.4	22.7	
CHA_2_DS_2_VASc risk score, median (IQR)	3 (2, 4)	4 (3, 5)	<0.001	4 (3, 5)	4 (3, 5)	<0.001
Cardiogenic shock, %	4.7	10.2	<0.001	6.2	11.0	<0.001
Cardiac arrest, %	2.6	4.6	<0.001	3.0	5.2	<0.001
Ventricular tachycardia, %	6.7	11.5	<0.001	8.2	11.2	<0.001
Ventricular fibrillation, %	3.4	5.7	<0.001	3.7	4.5	0.002
Pericardial effusion, %	0.5	1.3	<0.001	1.2	2.1	<0.001
Comorbidities, %						
Dyslipidemia	72.3	70.0	<0.001	67.6	62.8	<0.001
Thrombocytopenia	2.6	5.8	<0.001	7.6	10.3	<0.001
Coagulopathy	3.5	7.8	<0.001	9.6	13.8	<0.001
Anemias	11.7	21.9	<0.001	26.9	35.5	<0.001
Smoking	2.3	1.2	<0.001	1.5	1.2	0.010
Valvular disease	7.2	14.7	<0.001	10.1	15.2	<0.001
Chronic pulmonary disease	17.9	25.8	<0.001	26.5	29.9	<0.001
Chronic renal failure	16.7	30.9	<0.001	25.1	33.6	<0.001
Dementia	2.0	4.3	<0.001	2.6	3.9	<0.001
Homelessness	0.2	0.1	<0.001	0.1	0.1	0.816
Liver disease	1.8	2.4	<0.001	2.8	2.6	0.355
Metastatic disease	/	/		20.9	20.8	0.889
Bed size of hospital, %			<0.001			<0.001
small	13.7	13.2		12.0	13.8	
medium	29.1	29.0		28.4	27.6	
large	57.2	57.8		59.6	58.7	
Hospital Region, %			<0.001			<0.001
Northeast	19.1	18.6		21.0	19.9	
Midwest	25.3	27.2		25.8	29.5	
South	42.1	40.9		39.8	36.8	
West	13.4	13.4		13.4	13.8	
Location/teaching status of hospital, %			<0.001			0.198
rural	5.9	5.6		5.0	5.2	
urban non-teaching	23.3	22.6		21.5	20.6	
urban teaching	70.8	71.8		73.6	74.2	

*Abbreviations: AF – atrial fibrillation; IQR – interquartile range; CHA2DS2VASc risk score – risk score composed of the following components: congestive heart failure, arterial hypertension, age cut-offs (65-75 and ≥75 years), diabetes mellitus, previous stroke, vascular disease, and sex category.

### Baseline characteristics

In the non-cancer group, patients with AF were more likely to be older and have a higher CHA_2_DS_2_VASc risk score compared with patients without AF. They also had a significantly higher prevalence of cardiogenic shock, cardiac arrest, ventricular tachycardia (VT) and fibrillation (VF), pericardial effusion, thrombocytopenia, coagulopathy, anemia, chronic lung disease, chronic renal failure, and valvular disease. However, patients without AF were more likely to have dyslipidemia and to be smokers (Table 1).

In the cancer group, both patients with and without AF had similar CHA_2_DS_2_VASc risk score. Cancer patients with AF more frequently had the other aforementioned comorbidities, except for dyslipidemia and smoking, which were higher in patients without AF (Table 1). In all study groups, most patients had a moderate-high CHA_2_DS_2_VASc risk score (non-cancer group without AF: 89%; non-cancer group with AF: 97%; cancer group without AF: 95%; cancer group with AF: 97%) (Supplemental Figure 3(Supplementary figure 3)).

### Unadjusted outcomes

In both non-cancer and cancer groups, patients with AF compared with patients without AF had a significantly higher proportion of deaths (5.2% vs 2.3% in the non-cancer group, and 7.5% vs 4.6% in the cancer group, *P* < 0.001 for both), MACCE (7.3% vs 3.6% in the non-cancer group, and 9.5% vs 6.4% in the cancer group, *P* < 0.001 for both), and major bleeding (2.8% vs 1.3% in the non-cancer group, and 5.0% vs 3.0% in the cancer group, *P* < 0.001 for both). In the non-cancer group, acute ischemic stroke was significantly more common in patients with AF (1.8% vs 0.9%, *P* < 0.001). In the cancer group, this outcome was not significantly associated with AF presence (Table 2 and Supplemental Figure 4(Supplementary figure 4)).

**Table 2:** Comparison of unadjusted in-hospital clinical outcomes according to cancer and atrial fibrillation diagnosis*

Characteristics	Non-cancer group		*P*-value	Cancer group		*P*-value
	without AF (82.1%)	with AF (15.4%)		without AF (1.9%)	with AF (0.6%)	
All-cause mortality, %	2.3	5.2	<0.001	4.6	7.5	<0.001
MACCE, %	3.6	7.3	<0.001	6.4	9.5	<0.001
Acute ischemic stroke, %	0.9	1.8	<0.001	1.5	1.8	0.190
Major bleeding, %	1.3	2.8	<0.001	3.0	5.0	<0.001
Length of stay (days), median (IQR)	2 (2, 4)	4 (2, 7)	<0.001	3 (2, 6)	5 (3, 9)	<0.001
Total charges (USD), median (IQR)	80 044 (56 651, 119 637)	98 296 (65 466, 159 168)	<0.001	90 648 (61 332, 142 118)	110 461 (71 499, 180 892)	<0.001

*Abbreviations: AF – atrial fibrillation; IQR – interquartile range; MACCE – major adverse cardiovascular and cerebrovascular events (composite of all-cause mortality, ischemic stroke and reinfarction); USD – United States Dollar.

### Adjusted outcomes

**Figure 1:**
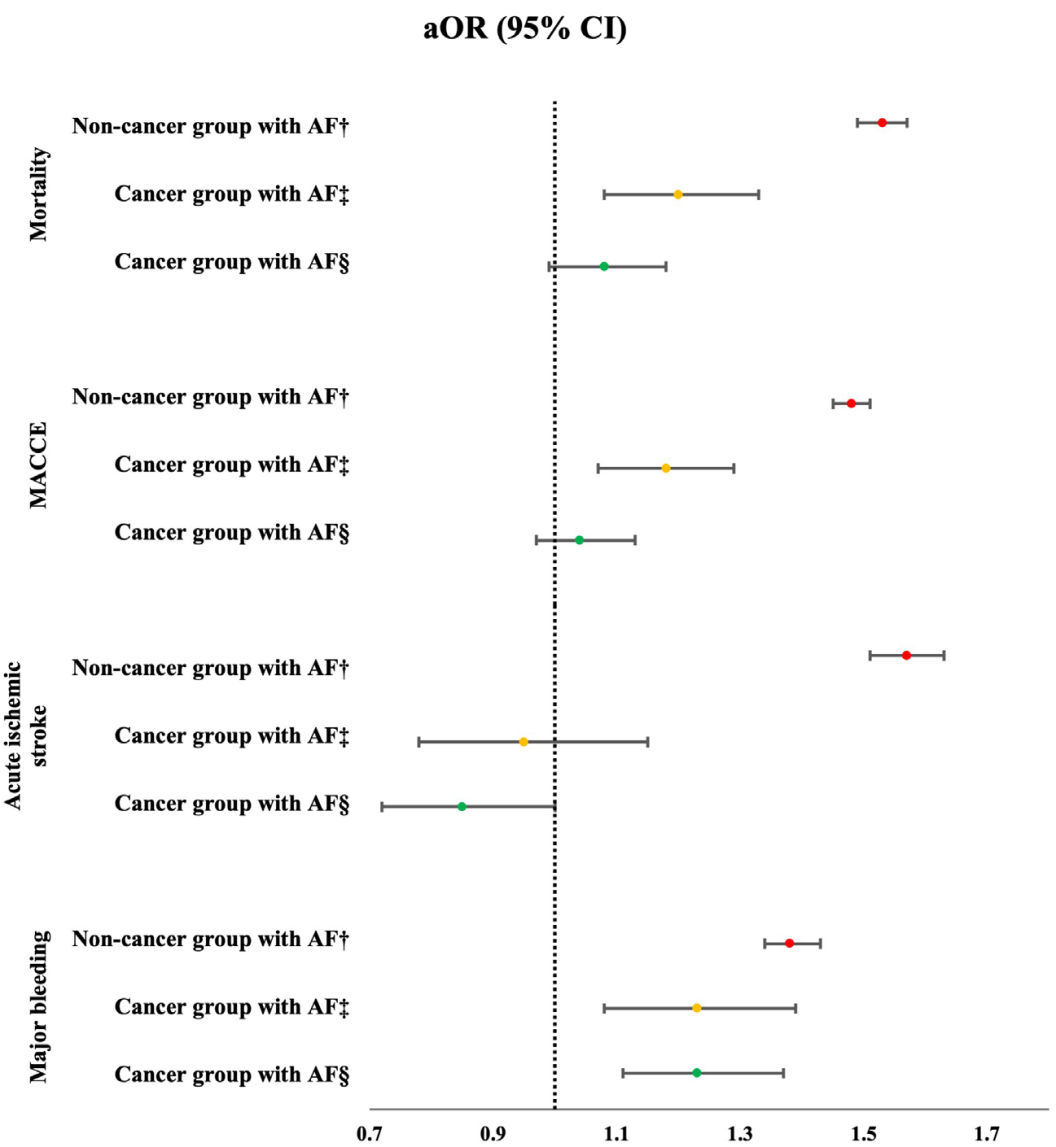
**Comparison of in-hospital clinical outcomes (adjusted outcomes).** Multivariable analysis – adjustment was done for the following variables: bed size of hospital, region of hospital, location/teaching status of hospital, age, sex, weekend admission, primary expected payer, smoking status, previous myocardial infarction, previous coronary artery bypass graft surgery, previous percutaneous coronary intervention, previous cerebrovascular accident, dementia, dyslipidemia, thrombocytopenia, and other comorbidities (anemias, chronic pulmonary disease, coagulopathy, diabetes mellitus, hypertension, liver disease, chronic renal failure). ^†^Reference group is non-cancer group without AF; ^‡^reference group is cancer group without AF; ^§^reference group is non-cancer group with AF. Abbreviations: AF – atrial fibrillation; aOR – adjusted odds ratios; CI – confidence interval; MACCE – major adverse cardiovascular and cerebrovascular events (composite of all-cause mortality, ischemic stroke, and reinfarction).

In multivariable logistic regression analysis, non-cancer patients with AF were more likely to develop in-hospital mortality, MACCE, acute ischemic stroke, and major bleeding compared with non-cancer patients without AF (aOR 1.53, 95% CI 1.49-1.57; aOR 1.48, 95% CI 1.45-1.51; aOR 1.57, 95% CI 1.51-1.63; and aOR 1.38, 95% CI 1.34-1.43, respectively, *P* < 0.001 for all). Cancer patients with AF were consistently more likely to develop all-cause mortality, MACCE, and major bleeding (aOR 1.20, 95% CI 1.08-1.33; aOR 1.18, 95% CI 1.07-1.29; and aOR 1.23, 95% CI 1.08-1.39, respectively) compared with cancer patients without AF, but there was no difference in acute ischemic stroke (*P* = 0.604). Compared with the non-cancer group with AF, cancer patients with AF only had significantly increased risk of major bleeding (aOR 1.23, 95% CI 1.11-1.37) (Table 3 and Figure 1).

**Table 3:** Comparison of adjusted^¶^ in-hospital clinical outcomes according to cancer and atrial fibrillation diagnosis*

Clinical outcomes	Non-cancer group with AF^†^		Cancer group with AF^‡^		Cancer group with AF^§^	
	aOR (95% CI)	*P*-value	aOR (95% CI)	*P*-value	aOR (95% CI)	*P*-value
All-cause mortality	1.53 (1.49, 1.57)	<0.001	1.20 (1.08, 1.33)	0.001	1.08 (0.99, 1.18)	0.096
MACCE	1.48 (1.45, 1.51)	<0.001	1.18 (1.07, 1.29)	0.001	1.04 (0.97, 1.13)	0.289
Acute ischemic stroke	1.57 (1.51, 1.63)	<0.001	0.95 (0.78, 1.15)	0.604	0.85 (0.72, 1.00)	0.055
Major bleeding	1.38 (1.34, 1.43)	<0.001	1.23 (1.08, 1.39)	0.001	1.23 (1.11, 1.37)	<0.001

*Abbreviations: AF – atrial fibrillation; aOR – adjusted odds ratios; CI – confidence interval; MACCE – major adverse cardiovascular and cerebrovascular events (composite of all-cause mortality, ischemic stroke, and reinfarction).

^†^Reference group is non-cancer group without AF.

^‡^Reference group is cancer group without AF.

^§^Reference group is non-cancer group with AF.

**¶**Multivariable analysis – adjustment was done for the following variables: bed size of hospital, region of hospital, location/teaching status of hospital, age, sex, weekend admission, primary expected payer, smoking status, previous myocardial infarction, previous coronary artery bypass graft surgery, previous percutaneous coronary intervention, previous cerebrovascular accident, dementia, dyslipidaemia, thrombocytopenia, and other comorbidities (anemias, chronic pulmonary disease, coagulopathy, diabetes mellitus, hypertension, liver disease, chronic renal failure).

### Sensitivity analyses

**Figure 2:**
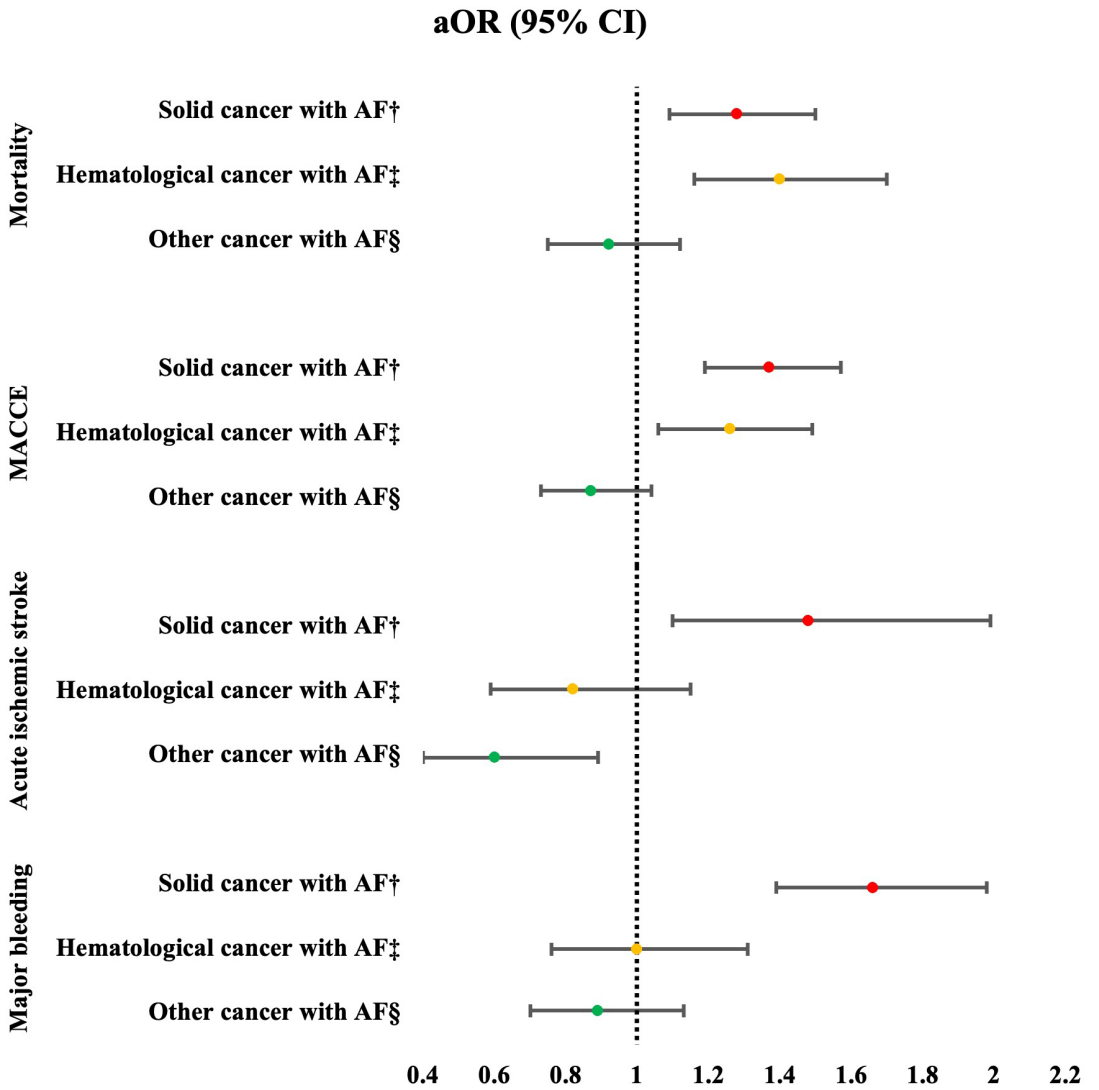
**Comparison of in-hospital clinical outcomes (adjusted outcomes) by cancer type.** Multivariable analysis – adjustment was done for the following variables: bed size of hospital, region of hospital, location/teaching status of hospital, age, sex, weekend admission, primary expected payer, smoking status, previous myocardial infarction, previous coronary artery bypass graft surgery, previous percutaneous coronary intervention, previous cerebrovascular accident, dementia, dyslipidemia, thrombocytopenia, and other comorbidities (anemias, chronic pulmonary disease, coagulopathy, diabetes mellitus, hypertension, liver disease, chronic renal failure). ^†^Reference group is solid cancer group without AF; ^‡^Reference group is hematological cancer group without AF; ^§^Reference group is other cancer group without AF. Abbreviations: AF – atrial fibrillation; aOR – adjusted odds ratios; CI – confidence interval; MACCE – major adverse cardiovascular and cerebrovascular events (composite of all-cause mortality, ischemic stroke and reinfarction).

Compared with cancer patients without AF, patients with AF, regardless of cancer type, had a higher proportion of any of the investigated outcomes, except for acute ischemic stroke, which was more common in the “other cancer” group without AF (Table 4 and Supplemental Figure 5(Supplementary figure 5)). After multivariable adjustment, solid cancer patients with AF had a higher aOR of all outcomes, including mortality (aOR 1.28, 95% CI 1.09-1.50), MACCE (aOR 1.37, 95% CI 1.19-1.57), acute ischemic stroke (aOR 1.48, 95% CI 1.10-1.99), and major bleeding (aOR 1.66, 95% CI 1.39-1.98) compared with the solid cancer group without AF. Furthermore, in patients with hematological cancer, the presence of AF was associated with increased all-cause mortality (aOR 1.40, 95% CI 1.16-1.70) and MACCE (aOR 1.26, 95% CI 1.06-1.49) but not with acute ischemic stroke and major bleeding (Table 5 and Figure 2). Unadjusted rates of clinical outcomes were further determined after stratification by exact subtype of solid cancer (Supplemental Table 2(Supplementary Table 2)). In particular, AF presence was associated with increased risk of stroke in patients with colorectal, breast, and prostate cancers, whereas the risk of stroke was similar in lung cancer patients with and without AF (Supplemental Table 3(Supplementary Table 3)).

**Table 4:** Comparison of unadjusted in-hospital clinical outcomes according to cancer and atrial fibrillation diagnosis, by cancer type*

Characteristics	Non-cancer group		Cancer group						*P*-value
			solid cancer		hematologic cancer		other cancer		
	without AF (82.1%)	with AF (15.4%)	without AF (0.7%)	with AF (0.2%)	without AF (0.6%)	with AF (0.2%)	without AF (0.6%)	with AF (0.2%)	
Number of hospitalizations	1 139 230	213 595	10 345	3435	8235	2430	7665	2385	
All-cause mortality, %	2.3	5.2	4.8	7.7	3.9	7.8	5.1	6.7	<0.001
MACCE, %	3.6	7.3	6.3	10.2	5.8	9.9	7.1	8.2	<0.001
Acute ischemic stroke, %	0.9	1.8	1.2	2.0	1.8	1.9	1.8	1.3	<0.001
Major bleeding, %	1.3	2.8	3.3	6.6	2.2	3.3	3.6	4.4	<0.001
Length of stay (days), median (IQR)	2 (2, 4)	4 (2, 7)	3 (2, 6)	5 (3, 9)	3 (2, 6)	5 (3, 9)	3 (2, 7)	6 (3, 10)	<0.001
Total charges (USD), median (IQR)	80 044 (56 651, 119 637)	98 296 (65 466, 159 168)	89 333 (61 332, 141 395)	109 658 (70 293, 173 910)	88 395 (60 461, 138 445)	113 991 (71 519, 178 535)	95 150 (62 599, 148 970)	109 690 (72 870, 202 298)	<0.001

*Abbreviations: AF – atrial fibrillation; IQR – interquartile range; MACCE – major adverse cardiovascular and cerebrovascular events (composite of all-cause mortality, ischemic stroke, and reinfarction); USD – Unites States Dollar.

**Table 5:** Comparison of adjusted** in-hospital clinical outcomes in cancer patients with atrial fibrillation stratified by cancer type*

Clinical outcomes	Non-cancer group with AF^†^		Cancer group with AF					
			solid cancer with AF^‡^		hematological cancer with AF^§^		other cancer with AF^¶^	
	aOR (95% CI)	*P*-value	aOR (95% CI)	*P*-value	aOR (95% CI)	*P*-value	aOR (95% CI)	*P*-value
All-cause mortality	1.53 (1.49, 1.57)	<0.001	1.28 (1.09, 1.50)	0.002	1.40 (1.16, 1.70)	0.001	0.92 (0.75, 1.12)	0.404
MACCE	1.48 (1.45, 1.51)	<0.001	1.37 (1.19, 1.57)	<0.001	1.26 (1.06, 1.49)	0.007	0.87 (0.73, 1.04)	0.119
Acute ischemic stroke	1.57 (1.51, 1.63)	<0.001	1.48 (1.10, 1.99)	0.010	0.82 (0.59, 1.15)	0.256	0.60 (0.40, 0.89)	0.011
Major bleeding	1.38 (1.34, 1.43)	<0.001	1.66 (1.39, 1.98)	<0.001	1.00 (0.76, 1.31)	0.999	0.89 (0.70, 1.13)	0.346

*Abbreviations: AF – atrial fibrillation; aOR – adjusted odds ratios; CI – confidence interval; MACCE – major adverse cardiovascular and cerebrovascular events (composite of all-cause mortality, ischemic stroke and reinfarction);

^†^Reference group is non-cancer group without AF.

^‡^Reference group is solid cancer group without AF.

^§^Reference group is hematological cancer group without AF.

^¶^Reference group is other cancer group without AF.

**Multivariable analysis – adjustment was done for the following variables: bed size of hospital, region of hospital, location/teaching status of hospital, age, sex, weekend admission, primary expected payer, smoking status, previous myocardial infarction, previous coronary artery bypass graft surgery, previous percutaneous coronary intervention, previous cerebrovascular accident, dementia, dyslipidaemia, thrombocytopenia, and other comorbidities (anemias, chronic pulmonary disease, coagulopathy, diabetes mellitus, hypertension, liver disease, chronic renal failure).

In the non-cancer group with AF, the findings were almost consistent with the overall cohort irrespective of the CHA_2_DS_2_VASc risk score. Notably, the presence of AF in patients who had a low CHA_2_DS_2_VASc risk score was associated with a greater aOR of all-cause mortality (aOR 4.88, 95% CI 2.21-10.79) and MACCE (aOR 3.49, 95% CI 1.73-7.05) compared with the same patients without AF (Table 6). Furthermore, in cancer patients who had a moderate-high CHA_2_DS_2_VASc risk score, AF was associated with increased risk of all-cause mortality (aOR 1.25, 95% CI 1.12-1.39), MACCE (aOR 1.22, 95% CI 1.11-1.34), and major bleeding (aOR 1.32, 95% CI 1.16-1.50) (Supplemental Table 4(Supplementary Table 4)).

**Table 6:** Comparison of adjusted** in-hospital clinical outcomes in non-cancer patients according to atrial fibrillation diagnosis, by thromboembolic risk (CHA_2_DS_2_VASc score)*

Clinical outcomes	CHA_2_DS_2_VASc risk score	Non-cancer group with AF^†^	
		aOR (95% CI)	*P*-value
All-cause mortality	**Low risk^‡^**	4.88 (2.21, 10.79)	<0.001
	**Low-moderate risk^§^**	2.20 (1.90, 2.55)	<0.001
	**Moderate-high risk^¶^**	1.53 (1.50, 1.57)	<0.001
MACCE	**Low risk^‡^**	3.49 (1.73, 7.05)	<0.001
	**Low-moderate risk^§^**	1.99 (1.75, 2.27)	<0.001
	**Moderate-high risk^¶^**	1.48 (1.45, 1.51)	<0.001
Acute ischemic stroke	**Low risk^‡^**	n/a	n/a
	**Low-moderate risk^§^**	1.53 (1.15, 2.03)	0.003
	**Moderate-high risk^¶^**	1.59 (1.52, 1.65)	<0.001
Major bleeding	**Low risk^‡^**	2.36 (0.86, 6.48)	0.095
	**Low-moderate risk^§^**	1.98 (1.61, 2.45)	<0.001
	**Moderate-high risk^¶^**	1.38 (1.34, 1.43)	<0.001

Abbreviations: n/a – unable to be evaluated due to low number of events; AF – atrial fibrillation; aOR – adjusted odds ratios; CHA_2_DS_2_VASc – risk score composed of the following components: congestive heart failure, arterial hypertension, age cut-offs (65-75 and ≥75 years), diabetes mellitus, previous stroke, vascular disease and sex category; CI – confidence interval; MACCE – major adverse cardiovascular and cerebrovascular events (composite of all-cause mortality, ischemic stroke, and reinfarction).

^†^Reference group is non-cancer group without AF.

^‡^CHA_2_DS_2_VASc = 0 in men; CHA_2_DS_2_VASc = 1 in women.

^§^CHA_2_DS_2_VASc = 1 in men; CHA_2_DS_2_VASc = 2 in women.

^¶^CHA_2_DS_2_VASc ≥2 in men; CHA_2_DS_2_VASc ≥3 in women.

**Multivariable analysis – adjustment was done for the following variables: bed size of hospital, region of hospital, location/teaching status of hospital, age, sex, weekend admission, primary expected payer, smoking status, dementia, dyslipidaemia, thrombocytopenia, and other comorbidities (anemias, chronic pulmonary disease, coagulopathy, liver disease, chronic renal failure).

When evaluating outcomes by PCI indication, we found that 425 535 patients had ST elevation myocardial infarction, 570 625 patients had non-ST-elevation myocardial infarction-acute coronary syndrome, and 391 160 patients had chronic coronary syndrome. Additionally, in cancer patients with AF compared with cancer patients without AF, irrespective of the PCI indication, aORs of mortality and MACCE were increased, whereas acute ischemic stroke risk was similar (Supplemental Table 5(Supplementary Table 5)).

## Discussion

In this retrospective nationwide study, we revealed outcomes of PCI patients according to the presence and type of cancer and AF diagnosis in a cohort of 1.3 million patients. Among cancer patients undergoing PCI, those with concomitant AF had a greater risk of mortality, MACCE, and major bleeding compared with cancer patients without AF, while the risk of stroke was similar between the two groups. In addition, compared with the non-cancer group with AF, patients with cancer and AF demonstrated a significantly increased risk of major bleeding. Notably, PCI outcomes in cancer patients with AF differed significantly according to type (solid and hematologic) and subtype of cancer (lung, prostate, breast, colorectal, and other cancer). Solid tumor patients with AF following PCI had a significantly higher risk of all-cause mortality, MACCE, acute ischemic stroke, and major bleeding, compared with solid tumor patients without AF. Patients with hematological cancer and AF had increased all-cause mortality and MACCE, while there was no difference in acute ischemic stroke and major bleeding compared with hematologic cancer without AF.

There are several shared etiopathologies between CAD, AF, and cancer. At an epidemiologic level, they share several risk factors including smoking, diabetes mellitus, obesity, and metabolic syndrome (12-14). Patients with cancer have a higher incidence and prevalence of AF compared with the general population, with one Danish population-based study reporting an incidence of AF of 17.4 per 1000 person-years in patients with cancer compared with 3.7 per 1000 person-years in patients without cancer (15). Additionally, cancer therapies are associated with the development of both AF and CAD. AF can occur in the post-operative setting after cancer resection (especially lung cancer) (16), as a result of chemotherapeutic agents [platinum-based chemotherapy (17), tyrosine kinase inhibitors (18)], or as a complication of cancer treatment such as neutropenia and infection (19). Likewise, chemotherapy, immunotherapy, and radiation for cancer accelerate atherosclerosis and may precipitate acute myocardial infarction (20). At a cellular level, inflammation plays a key role in the pathophysiology of cancer, AF, and CAD (21,22). Inflammatory markers such as high-sensitivity C-reactive protein and interleukin (IL)-6 are associated with both CAD and AF (23,24). Likewise, several inflammatory markers (such as IL-1 and IL-6) have been shown to be associated with the development and progression of cancer and have therefore also become targets for cancer treatment (25,26).

Numerous challenges are associated with performing PCI in patients with cancer and AF, which ultimately may impact clinical outcomes. Our current study shows that patients with cancer and AF undergoing PCI are older and have more comorbidities, such as chronic renal failure and pulmonary disease. They also tend to have more valvular heart disease and present more often with cardiogenic shock, ventricular tachycardia, and cardiac arrest. Additionally, they have a greater prevalence of anemia and thrombocytopenia. The Academic Research Consortium for High Bleeding Risk has identified active malignancy, anemia, and thrombocytopenia as independent major risk factors for bleeding at the time of PCI (27). Numerous previous studies have shown that cancer patients undergoing PCI are at increased risk of bleeding (28,29), and in-hospital (28) and long-term mortality (29,30).

Furthermore, patients with AF undergoing PCI often require triple therapy (dual antiplatelet plus oral anticoagulant) at least for the short-term, which further increases their risk of bleeding (31). Higher bleeding risk in patients with AF was also observed in our study, particularly in patients with colorectal cancer and AF, with observed in-hospital major bleeding rates of 11.9%. The high risk of major bleeding in patients with cancer and comorbid AF undergoing PCI would support the use of bleeding-avoidance strategies such as a radial first approach (32), use of direct oral anticoagulants (DOACs) instead of warfarin, or dual therapy (DOAC and single antiplatelet) for only 6 months as per guideline recommendations (33). Additionally, cancer patients undergoing PCI have also shown to be at a heightened risk for stent thrombosis (34) and repeat target vessel revascularization (35). Interruption of antiplatelet agents or oral anticoagulants for cancer surgery, biopsy, or chemotherapy-induced thrombocytopenia is also a concern for bleeding and thrombotic complications (36,37).

One of the major strengths of our study is that it highlights the differences in PCI outcomes according to the presence of both AF and cancer, whereas numerous previous studies explored the clinical outcomes of PCI among patients with cancer only or with AF only or outcomes of AF and cancer only (5,38-40). Additionally, we analyzed data from 1.3 million patients, which represents a larger sample than those used in the previous studies. We also stratified our analysis by type of cancer, whether solid or hematological, and further stratified the solid cancer group into lung, prostate, breast, and colon cancer.

In our study, cancer patients with AF did not have a significantly increased risk of acute ischemic stroke compared with cancer patients without AF. Indeed, in unadjusted analysis, acute ischemic stroke was significantly more common in patients with AF in the non-cancer cohort, but in the cancer group this outcome was not significantly associated with AF presence. Also, when looking at the adjusted rates, no significant differences were observed. There are several potential explanations. First, this finding emphasizes the complex interaction between various patient-related factors and ischemic stroke occurrence. Second, the NIS does not capture data on medications, including anticoagulants, that may decrease the stroke risk and increase bleeding risk in patients with AF. Third, this study encompassed only in-hospital outcomes, including stroke, so it does not correspond to overall or long-term stroke rates, which may be different. Moreover, we revealed important differences when stratifying the analysis by cancer type. Patients with solid cancer and AF had a higher risk of ischemic stroke than solid cancer patients without AF. However, interestingly, patients with hematological cancer and AF had a similar risk of stroke to hematological cancer patients without AF. Hematological cancer *per se* increases major bleeding and stroke risk substantially (41), so the addition of AF did not considerably further increase this risk. Furthermore, clonal hematopoiesis of indeterminate potential (CHIP), a pre-malignant state of hematologic malignancy, is fairly common in the elderly population. These patients have an increased cardiovascular risk and have a higher occurrence of AF than non-CHIP patients (42), similar to patients with fully established myeloproliferative diseases (43). There is probably a substantial proportion of CHIP patients in the non-cancer cohorts, which may potentially confound the analysis. It is difficult to interpret the results regarding the decreased risk of ischemic stroke in the “other cancer” group with AF compared with non-AF patients due to the heterogeneous cancers included and lacking data on cancer stage and treatments administered. However, it might be that the majority of patients with “other cancer” had a minimally increased baseline stroke risk but when diagnosed with AF, they were treated aggressively, which lowered the risk of stroke consequently. AF patients with solid tumor had significantly higher risk of all-cause mortality, MACCE, acute ischemic stroke, and major bleeding following PCI. Among the different types of solid tumors, patients with lung cancer with AF had the highest absolute risk of all-cause mortality and MACCE, while patients with colorectal and prostate cancer with AF had the highest risk of bleeding following PCI. These findings are consistent with our previous work, which demonstrated that patients with lung cancer undergoing PCI had the highest risk of in-hospital mortality, while those with colon cancer had the highest risk of bleeding (4). This once again underscores the fact that not all cancer patients can be grouped into one cohort, but they actually represent a heterogeneous group with varying outcomes and the risk of bleeding (44).

There are several limitations to our study that are inherent to the data set. First, since we used electronic health care data, miscoding could be present. Therefore, there is a possibility of potential error, although ICD-10 codes have been validated previously (45,46). In addition, the NIS lacks information about patients with high-risk coronary anatomy, left ventricular function, VT/VF pre-PCI, cancer stage, treatment, and management plans where the chosen antiplatelet and anticoagulant therapies could impact the outcomes in the study subgroups. We also could not discern the risk of mortality according to specific cause (cardiac/non-cardiac) as the NIS does not report a specific cause of death. Moreover, we could only investigate adverse events that occurred in hospital as the NIS does not include long-term outcomes.

In conclusion, patients with cancer and AF undergoing PCI have worse outcomes, including mortality, MACCE, and major bleeding compared with cancer patients without AF. However, clinical outcomes and bleeding risk vary significantly based on type of cancer. These findings suggest that among cancer patients with AF, the indication for PCI, and duration and choice of anti-platelet and oral anticoagulants should be carefully considered and tailored to the individual patient based on their bleeding and thrombotic risk. A shared decision-making with the involvement of the interventional cardiologist, oncologist, and the patient and family is recommended.
