# Supplementary material for: Outcomes of 1.3 million patients undergoing percutaneous coronary intervention according to the presence of cancer and atrial fibrillation: a retrospective study
Source: Croat Med J. 2024 Oct;65(5):405–16. doi: 10.3325/cmj.2024.65.405 (PMC11568383; doi:10.3325/cmj.2024.65.405)

Supplementary Figure 2. Prevalence of specific cancer types: **A.** Total cancer cohort; **B.** Cancer patients with atrial fibrillation.

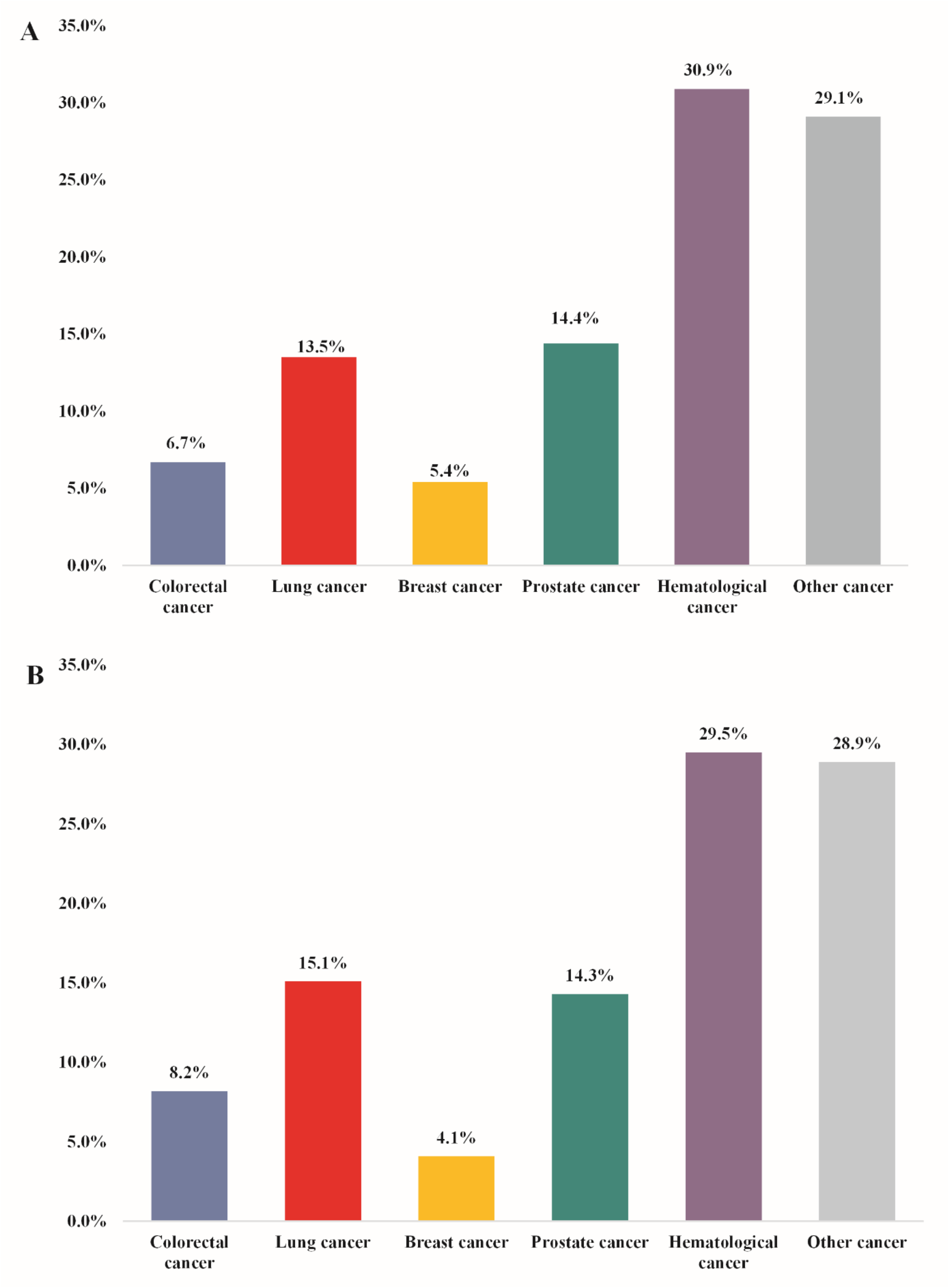

Supplement: Supplementary Figure 2 [file CroatMedJ_65_s002.pdf]
