# Supplementary material for: Outcomes of 1.3 million patients undergoing percutaneous coronary intervention according to the presence of cancer and atrial fibrillation: a retrospective study
Source: Croat Med J. 2024 Oct;65(5):405–16. doi: 10.3325/cmj.2024.65.405 (PMC11568383; doi:10.3325/cmj.2024.65.405)
Supplement: Supplementary Figure 3 [file CroatMedJ_65_s003.pdf]

Supplementary Figure 3. Total study population stratified by CHA2DS2VASc risk score according to cancer and AF presence.

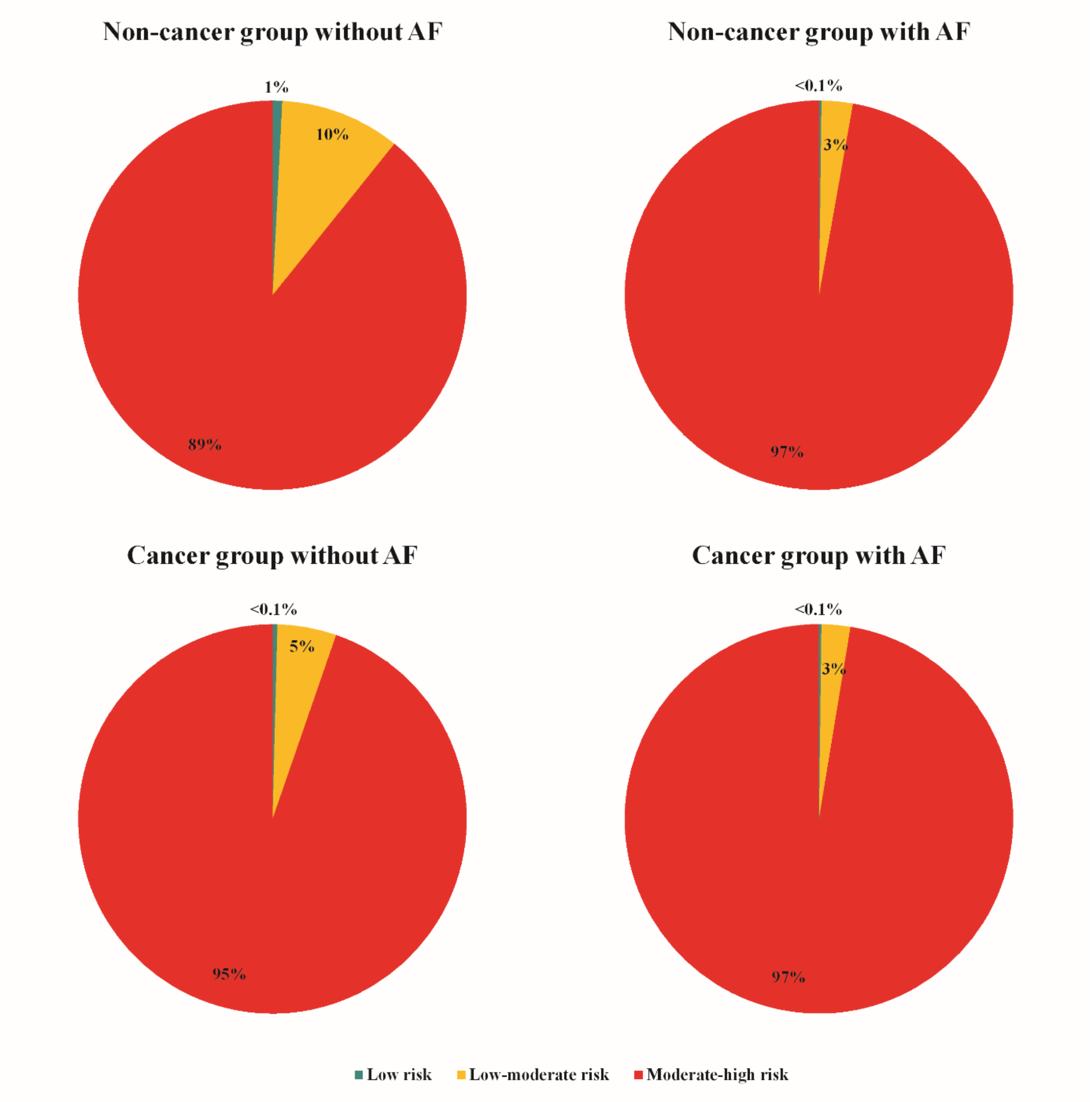

Abbreviations: AF – atrial fibrillation; CHA2DS2VASc risk score – risk score composed of the following components: congestive heart failure, arterial hypertension, age cut-offs (65-75 and ≥75 years), diabetes mellitus, previous stroke, vascular disease and sex category.

Notes: Low risk includes CHA2DS2VASc=0 in males; CHA2DS2VASc=1 in females. Low-moderate risk includes CHA2DS2VASc=1 in males; CHA2DS2VASc=2 in females. Moderate-high risk includes CHA2DS2VASc≥2 in males; CHA2DS2VASc≥3 in females.
