# Supplementary material for: Outcomes of 1.3 million patients undergoing percutaneous coronary intervention according to the presence of cancer and atrial fibrillation: a retrospective study
Source: Croat Med J. 2024 Oct;65(5):405–16. doi: 10.3325/cmj.2024.65.405 (PMC11568383; doi:10.3325/cmj.2024.65.405)
Supplement: Supplementary Figure 4 [file CroatMedJ_65_s004.pdf]

Supplementary Figure 4. Comparison of in-hospital clinical outcomes (unadjusted outcomes).

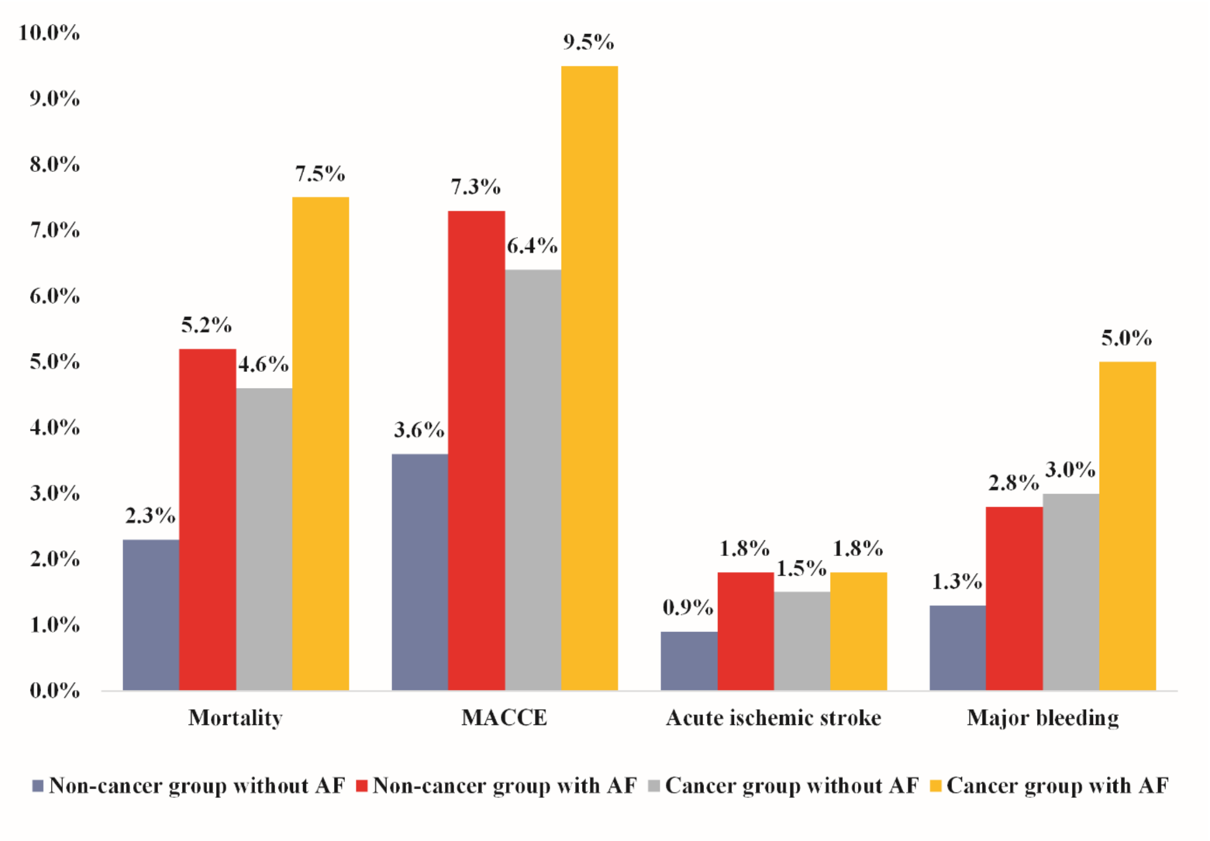

Abbreviations: AF – atrial fibrillation; MACCE – major adverse cardiovascular and cerebrovascular events (composite of all-cause mortality, ischemic stroke and reinfarction).
