# Supplementary material for: Outcomes of 1.3 million patients undergoing percutaneous coronary intervention according to the presence of cancer and atrial fibrillation: a retrospective study
Source: Croat Med J. 2024 Oct;65(5):405–16. doi: 10.3325/cmj.2024.65.405 (PMC11568383; doi:10.3325/cmj.2024.65.405)
Supplement: Supplementary Table 1 [file CroatMedJ_65_s006.pdf]

Supplementary Table 1. Search codes.

Diagnoses and outcomes	Source	Codes
**Atrial fibrillation**	ICD-10	I48.91, I48.20-21, I48.11, I48.19, I48.0
**Atrial flutter**	ICD-10	I48.3, I48.4, I48.92
**Cancer**	ICD-10	C00-C96
**Colorectal cancer**	ICD-10	C18-C21
**Lung cancer**	ICD-10	C34
**Breast cancer**	ICD-10	C50
**Prostate cancer**	ICD-10	C61
**Hematological cancer**	ICD-10	C81-C96
**Dyslipidaemia**	ICD-10	E78*
**Smoker**	ICD-10	Z72.0
**Cardiac arrest**	ICD-10	I46.2 (due to cardiac condition); I46.8 and I46.9 (due to non-cardiac condition)
**Heart Failure**	ICD-10	I50* Cardiomyopathy, I42*
**Ischemic cardiomyopathy**	ICD-10	I25.5
**Ventricular Tachycardia/Fibrillation**	ICD-10	VF: I49.01, I490.2; VT: I47.0, I47.2
**Previous CVA (TIA and Stroke)**	ICD-10	Z86.73
**Dementia (Presenile, Senile, Vascular and Alzheimer's)**	ICD-10	F01*, F02*, F03*
**Thrombocytopenia**	ICD-10	D69.4*, D69.5*, D69.6*
**Homelessness**	ICD-10	Z59.0
**Chronic renal failure**	ICD-10	N18*
**Hypertension**	ICD-10	I10*
**Anemias**	ICD-10	D62*, D63*, D64*
**Chronic Lung Disease (inc. bronchitis, COPD, asthma and bronchiectasis)**	ICD-10	J41*, J42*, J43*, J44*, J45*, J47*
**Diabetes Mellitus**	ICD-10	E08*, E09*, E10*, E11*, E13*
**Coagulopathies**	ICD-10	D65, D66, D67, D68*, D69*
**Liver disease**	ICD-10	K70*, K72.1*, K72.9*, K73*, K74*, K75*, K76*, K77*
**Metastatic disease**	ICD-10	C77*, C78*, C79*, R18.0*, C7B*
**Peripheral vascular disease**	ICD-10	I73*

**Valvular heart disease**	ICD-10	I34*, I35*, I36*, I37*
**Acute ischemic stroke**	ICD-10	I63*
**Reinfarction**	ICD-10	I22.x
**Major bleeding**	ICD-10	I60*, I61*, I62*, R58, K92.0, K92.1, K92.2
**Pericardial effusion**	ICD-10	I31.3
**CHA_2DS_2VASc score components**	**Source**	**Codes**
**Congestive heart failure**	ICD-10	I50*, I42*
**Arterial hypertension**	ICD-10	I10*, I110, I119, I12, I120, I129, I13, I130, I131, I1310, I1311, I132, I15, I150, I151, I152, I158, I159, I674
**Diabetes Mellitus**	ICD-10	E08*, E09*, E10*, E11*, E13*
**Previous Stroke**	ICD-10	Z8673
**Vascular disease**	ICD-10	I73*, I21, I20.0, I252, I256, Z9861, Z951, Z955

Abbreviations: CHA_2DS_2VASc risk score – risk score composed of the following components: congestive heart failure, arterial hypertension, age cut-offs (65-75 and ≥75 years), diabetes mellitus, previous stroke, vascular disease and sex category; COPD – chronic obstructive pulmonary disease; CVA – cerebrovascular accident; ICD-10 – International Classification of Diseases Tenth Edition; TIA – transient ischemic attack.
