# Supplementary material for: Outcomes of 1.3 million patients undergoing percutaneous coronary intervention according to the presence of cancer and atrial fibrillation: a retrospective study
Source: Croat Med J. 2024 Oct;65(5):405–16. doi: 10.3325/cmj.2024.65.405 (PMC11568383; doi:10.3325/cmj.2024.65.405)
Supplement: Supplementary Table 2 [file CroatMedJ_65_s007.pdf]

Supplementary Table 2. Comparison of unadjusted in-hospital clinical outcomes according to cancer and atrial fibrillation/flutter diagnosis, by specific cancer type.

Characteristics	Cancer cohort												*P*-value
	Colorectal cancer		Lung cancer		Breast cancer		Prostate cancer		Hematological cancer		Other cancer		
	Without AF (0.1%)	With AF (<0.1%)	Without AF (0.2%)	With AF (0.1%)	Without AF (0.1%)	With AF (<0.1%)	Without AF (0.3%)	With AF (0.1%)	Without AF (0.6%)	With AF (0.2%)	Without AF (0.6%)	With AF (0.2%)	
**Number of hospitalizations**	1,625	675	3,395	1,245	1,525	335	3,800	1,180	8,235	2,430	7,665	2,385	
**All-cause mortality**	6.2	5.9	8.0	12.0	3.9	3.0	1.8	5.5	3.9	7.8	5.1	6.7	<0.001
**MACCE**	7.4	7.4	10.2	14.9	5.6	6.0	2.8	8.1	5.8	9.9	7.1	8.2	<0.001
**Acute ischemic stroke**	0.6	2.2	2.2	1.6	0.7	3.0	0.8	2.1	1.8	1.9	1.8	1.3	<0.001
**Acute haemorrhagic stroke**	0.3	0.0	0.1	0.4	0.0	0.0	0.0	0.0	0.2	0.0	0.3	0.0	<0.001
**Major bleeding**	9.5	11.9	2.5	4.4	1.6	1.5	2.0	7.2	2.2	3.3	3.6	4.4	<0.001
**Length of stay (days), median (IQR)**	4 (2, 8)	5 (2, 11)	4 (2, 7)	6 (3, 9)	3 (2, 5)	5 (3, 8)	3 (2, 5)	4 (2, 9)	3 (2, 6)	5 (3, 9)	3 (2, 7)	6 (3, 10)	<0.001
**Total charges (USD), median (IQR)**	101,634 (64,982, 166,688)	115,185 (67,896, 200,003)	94,655 (66,101, 146,661)	109,460 (71,990, 163,726)	80,300 (58,923, 123,404)	113,687 (78,222, 201,270)	84,871 (57,995, 132,684)	105,828 (64,146, 162,798)	88,395 (60,461, 138,445)	113,991 (71,519, 178,535)	95,150 (62,599, 148,970)	109,690 (72,870, 202,298)	<0.001

Abbreviations: AF – atrial fibrillation/flutter; IQR – interquartile range; MACCE – major adverse cardiovascular and cerebrovascular events (composite of all-cause mortality, ischemic stroke and reinfarction); USD – Unites States Dollar.
