# Supplementary material for: Outcomes of 1.3 million patients undergoing percutaneous coronary intervention according to the presence of cancer and atrial fibrillation: a retrospective study
Source: Croat Med J. 2024 Oct;65(5):405–16. doi: 10.3325/cmj.2024.65.405 (PMC11568383; doi:10.3325/cmj.2024.65.405)
Supplement: Supplementary Table 3 [file CroatMedJ_65_s008.pdf]

Supplementary Table 3. Comparison of adjusted in-hospital clinical outcomes in cancer patients with AF stratified by cancer type.

**A. Clinical outcomes**	**Non-cancer group with AF[1]**		**Cancer group with AF**													
			**Colorectal cancer with AF[2]**		**Lung cancer with AF[3]**		**Breast cancer with AF[4]**		**Prostate cancer with AF[5]**		**Hematological cancer with AF[6]**		**Other cancer with AF[7]**	
	**aOR [95% CI]**	***P*-value**	**aOR [95% CI]**	***P*-value**	**aOR [95% CI]**	***P*-value**	**aOR [95% CI]**	***P*-value**	**aOR [95% CI]**	***P*-value**	**aOR [95% CI]**	***P*-value**	**aOR [95% CI]**	***P*-value**
**All-cause mortality**	1.53 [1.49, 1.57]	<0.001	0.77 [0.52, 1.13]	0.184	1.30 [1.05, 1.62]	0.018	0.47 [0.24, 0.94]	0.033	2.43 [1.71, 3.45]	<0.001	1.40 [1.16, 1.70]	0.001	0.92 [0.75, 1.12]	0.404
**MACCE**	1.48 [1.45, 1.51]	<0.001	0.84 [0.59, 1.19]	0.322	1.32 [1.08, 1.60]	0.006	0.73 [0.44, 1.22]	0.233	2.53 [1.90, 3.39]	<0.001	1.26 [1.06, 1.49]	0.007	0.87 [0.73, 1.04]	0.119
**Acute ischemic stroke**	1.57 [1.51, 1.63]	<0.001	3.26 [1.45, 7.31]	0.004	0.65 [0.39, 1.07]	0.089	3.49 [1.44, 8.50]	0.006	2.38 [1.39, 4.08]	0.002	0.82 [0.59, 1.15]	0.256	0.60 [0.40, 0.89]	0.011
**Major bleeding**	1.38 [1.34, 1.43]	<0.001	1.08 [0.80, 1.46]	0.622	1.50 [1.05, 2.13]	0.025	0.59 [0.22, 1.57]	0.294	3.25 [2.34, 4.51]	<0.001	1.00 [0.76, 1.31]	0.999	0.89 [0.70, 1.13]	0.346

[1]Reference group is non-cancer group without AF.
[2]Reference group is colorectal cancer group without AF.
[3]Reference group is lung cancer group without AF.
[4]Reference group is breast cancer group without AF.
[5]Reference group is prostate cancer group without AF.
[6]Reference group is hematological cancer group without AF.
[7]Reference group is other cancer group without AF.

Abbreviations: AF – atrial fibrillation/flutter; aOR – adjusted odds ratios; CI – confidence interval; MACCE – major adverse cardiovascular and cerebrovascular events (composite of all-cause mortality, ischemic stroke and reinfarction).

Multivariable analysis – adjustment was done for the following variables: bed size of hospital, region of hospital, location/teaching status of hospital, age, sex, weekend admission, primary expected payer, smoking status, previous myocardial infarction, previous coronary artery bypass graft surgery, previous percutaneous coronary intervention, previous cerebrovascular accident, dementia, dyslipidaemia, thrombocytopenia, and other comorbidities (anaemias, chronic pulmonary disease, coagulopathy, diabetes mellitus, hypertension, liver disease, chronic renal failure).
