# Supplementary material for: Outcomes of 1.3 million patients undergoing percutaneous coronary intervention according to the presence of cancer and atrial fibrillation: a retrospective study
Source: Croat Med J. 2024 Oct;65(5):405–16. doi: 10.3325/cmj.2024.65.405 (PMC11568383; doi:10.3325/cmj.2024.65.405)
Supplement: Supplementary Table 4 [file CroatMedJ_65_s009.pdf]

Supplementary Table 4. Comparison of adjusted in-hospital clinical outcomes in cancer patients according and atrial fibrillation/flutter diagnosis, by thromboembolic risk (CHA_2DS_2VASc score).

A. Clinical outcomes	CHA_2DS_2VASc risk score	Cancer group with AF[1]	
		aOR [95% CI]	*P*-value
**All-cause mortality**	**Low risk[2]**	n/a*	n/a*
	**Low-moderate risk[3]**	0.70 [0.34, 1.42]	0.321
	**Moderate-high risk[4]**	1.25 [1.12, 1.39]	<0.001
**MACCE**	**Low risk[2]**	n/a*	n/a*
	**Low-moderate risk[3]**	0.78 [0.43, 1.42]	0.420
	**Moderate-high risk[4]**	1.22 [1.11, 1.34]	<0.001
**Acute ischemic stroke**	**Low risk[2]**	n/a*	n/a*
	**Low-moderate risk[3]**	0.71 [0.26, 1.92]	0.503
	**Moderate-high risk[4]**	0.98 [0.80, 1.19]	0.82
**Major bleeding**	**Low risk[2]**	n/a*	n/a*
	**Low-moderate risk[3]**	0.27 [0.10, 0.71]	0.008
	**Moderate-high risk[4]**	1.32 [1.16, 1.50]	<0.001

[1]Reference group is cancer group without AF.

[2]$CHA_2DS_2VASc=0$ in males; $CHA_2DS_2VASc=1$ in females.

[3]$CHA_2DS_2VASc=1$ in males; $CHA_2DS_2VASc=2$ in females.

[4]$CHA_2DS_2VASc\geq2$ in males; $CHA_2DS_2VASc\geq3$ in females.

Abbreviations: *Not applicable - unable to be evaluated due to low number of events; AF – atrial fibrillation/flutter; aOR – adjusted odds ratios; CHA_2DS_2VASc risk score – risk score composed of the following components: congestive heart failure, arterial hypertension, age cut-offs (65-75 and ≥75 years), diabetes mellitus, previous stroke, vascular disease and sex category; CI – confidence interval; MACCE – major adverse cardiovascular and cerebrovascular events (composite of all-cause mortality, ischemic stroke and reinfarction).

Multivariable analysis – adjustment was done for the following variables: bed size of hospital, region of hospital, location/teaching status of hospital, age, sex, weekend admission, primary expected payer, smoking status, dementia, dyslipidaemia, thrombocytopenia, and other comorbidities (anaemias, chronic pulmonary disease, coagulopathy, liver disease, chronic renal failure).
