# Supplementary material for: Outcomes of 1.3 million patients undergoing percutaneous coronary intervention according to the presence of cancer and atrial fibrillation: a retrospective study
Source: Croat Med J. 2024 Oct;65(5):405–16. doi: 10.3325/cmj.2024.65.405 (PMC11568383; doi:10.3325/cmj.2024.65.405)
Supplement: Supplementary Table 5 [file CroatMedJ_65_s010.pdf]

Supplementary Table 5. Comparison of adjusted in-hospital clinical outcomes according to cancer and atrial fibrillation/flutter diagnosis, by PCI indication.

B. Clinical outcomes	PCI indication	Cancer group with AF[1]	
		aOR [95% CI]	*P*-value
**All-cause mortality**	**STEMI**	1.17 [1.00, 1.35]	0.045
	**NSTE-ACS**	1.42 [1.18, 1.69]	<0.001
	**CCS**	1.42 [1.07, 1.90]	0.016
**MACCE**	**STEMI**	1.21 [1.06, 1.39]	0.006
	**NSTE-ACS**	1.30 [1.12, 1.51]	0.001
	**CCS**	1.28 [1.01, 1.63]	0.046
**Acute ischemic stroke**	**STEMI**	0.98 [0.74, 1.31]	0.891
	**NSTE-ACS**	0.96 [0.69, 1.33]	0.792
	**CCS**	1.05 [0.68, 1.63]	0.822
**Major bleeding**	**STEMI**	1.39 [1.12, 1.72]	0.003
	**NSTE-ACS**	1.42 [1.18, 1.72]	<0.001
	**CCS**	0.82 [0.63, 1.07]	0.148

[1]Reference group is the same cancer group without AF.

Abbreviations: AF – atrial fibrillation/flutter; aOR – adjusted odds ratios; CCS – chronic coronary syndrome; CI – confidence interval; MACCE – major adverse cardiovascular and cerebrovascular events (composite of all-cause mortality, ischemic stroke and reinfarction); NSTE-ACS – non-ST-elevation myocardial infarction – acute coronary syndrome; STEMI – ST-elevation myocardial infarction.

Multivariable analysis – adjustment was done for the following variables: bed size of hospital, region of hospital, location/teaching status of hospital, age, sex, weekend admission, primary expected payer, smoking status, previous myocardial infarction, previous coronary artery bypass graft surgery, previous percutaneous coronary intervention, previous cerebrovascular accident, dementia, dyslipidaemia, thrombocytopenia, and other comorbidities (anaemias, chronic pulmonary disease, coagulopathy, diabetes mellitus, hypertension, liver disease, chronic renal failure
